# The Architecture of Deep Phenotyping in Asthma: Integrating Molecular, Metabolic, and Neuro-Hormonal Endotypes

**DOI:** 10.3390/ijms27062545

**Published:** 2026-03-10

**Authors:** Nicolae Demenciuc, Corina Ureche, Corina Eugenia Budin, Mircea Stoian, Teodora Nicola-Varo, Edith Simona Ianosi, Dariana-Elena Pătrîntașu, Anca Goman, Lavinia Davidescu, Diana Deleanu

**Affiliations:** 1 Department of Allergology, George Emil Palade University of Medicine, Pharmacy, Science and Technology of Târgu Mureș, 540142 Târgu Mureș, Romania; demenciuc_nicolae@yahoo.com (N.D.);; 2 Doctoral School, George Emil Palade University of Medicine, Pharmacy, Science and Technology of Târgu Mureș, 540142 Târgu Mureș, Romania; 3 Intensive Care Unit, Emergency County Hospital Târgu Mureș, 540136 Târgu Mureș, Romania; 4 Department of Allergology, Emergency County Hospital Târgu Mureș, 540136 Târgu Mureș, Romania; 5 Department of Pneumology, Mureș Clinical County Hospital, 540003 Târgu Mureș, Romania; 6 Department of Pathophysiology, George Emil Palade University of Medicine, Pharmacy, Science and Technology of Târgu Mureș, 540142 Târgu Mureș, Romania; 7 Department of Anesthesiology and Intensive Care, George Emil Palade University of Medicine, Pharmacy, Science and Technology of Târgu Mureș, 540142 Târgu Mureș, Romania; 8 Intensive Care Unit, Mures Clinical Country Hospital, Street Gheorghe Marinescu No.1, 540103 Târgu Mureș, Romania; 9 Department of Pulmonology, University of Medicine, Pharmacy, Science and Technology “George Emil Palade” of Târgu Mureș, 540139 Târgu Mureș, Romania; 10 Department of Pulmonology, Doctoral School of Biomedical Sciences, University of Oradea, 410073 Oradea, Romania; 11 Department of Pneumology, Faculty of Medicine and Pharmacy, University of Oradea, 410073 Oradea, Romania

**Keywords:** severe asthma, precision medicine, biomarkers, endotyping, airway remodeling

## Abstract

Asthma is increasingly recognized as a heterogeneous syndrome where traditional management fails, particularly given spirometry’s limitations in assessing small airway dysfunction. This review synthesizes the transition from clinical phenotyping to deep molecular endotyping, establishing a framework for precision medicine. We highlight the insufficiency of absolute eosinophil counts, proposing eosinophil cationic protein (ECP) and eosinophil-derived neurotoxin (EDN) as superior activation metrics. Furthermore, we explore Type 2 drivers (IL-4/IL-13, periostin) and epithelial alarmins like TSLP. Beyond classical immunology, the text describes metabolic dysregulation, specifically asymmetric dimethylarginine (ADMA) in obese-asthma phenotypes where nitric oxide synthase uncoupling promotes oxidative stress. We also analyze YKL-40 and surfactant protein D (SP-D) as markers of remodeling and barrier permeability, alongside microRNAs—specifically miR-21—in corticosteroid resistance. We conclude that managing refractory asthma requires shifting from reactive symptom control to an integrated analysis of multi-omic biomarkers. Establishing this comprehensive molecular profile via specialized centers is fundamental for addressing current diagnostic limitations, selecting biological therapies, and modifying the disease trajectory through an endotype-driven strategy addressing inflammatory, metabolic, and structural pathologies.

## 1. Introduction: The Imperative for Deep Phenotyping in Asthma

### 1.1. Limitations of Standard Spirometry in the Era of Precision Medicine

Asthma is no longer viewed as a single disease entity but as a complex, heterogeneous syndrome characterized by diverse underlying pathophysiological mechanisms [1]. In the era of precision medicine, the traditional “one-size-fits-all” approach has been replaced by a need for deep phenotyping, a process that integrates clinical, functional, and molecular data to define specific patient clusters [2]. Although standard spirometry remains the primary diagnostic tool, its reliance on the forced expiratory volume in one second (FEV1) offers only a surrogate measure of large airway patency, often failing to capture the complex involvement of the distal lung compartment [3].

Clinical practice frequently encounters a functional–symptomatic dissociation, where patients exhibit near-normal spirometric values despite persistent symptoms and ongoing airway remodeling. This limitation underscores the diagnostic gap of conventional lung function tests regarding the small airways (those < 2 mm in diameter), which are often the primary site of inflammation in severe phenotypes [4].

Consequently, small airway disease (SAD) must be explicitly conceptualized not merely as a distal anatomical region, but as a dynamic immunometabolic interface. It is precisely within this distal compartment that Type 2 inflammation, ongoing epithelial injury, oxidative stress, and structural remodeling converge [5]. Because peripheral airway inflammation frequently persists despite relatively preserved or normalized conventional spirometric indices, relying exclusively on FEV1 masks highly biologically active disease compartments [6]. Integrating SAD into a multi-omic endotyping model provides a far more robust mechanistic foundation for the precision strategies proposed herein.

Beyond mechanics, the identification of specific endotypes—such as Th2 and non-Th2 inflammation—requires the integration of a broad spectrum of biomarkers. By bridging the gap between systemic immune signals, hormonal modulators, and lung mechanics, we can transition from a reactive treatment model to a proactive, endotype-driven therapeutic strategy [7].

### 1.2. The Transition from Clinical Phenotyping to Molecular Endotyping

The paradigm shift from clinical phenotyping to molecular endotyping represents a key step in the modernization of asthma management. While clinical phenotypes describe observable characteristics such as exacerbation frequency or age of onset, they often fail to explain the underlying biological mechanisms (endotypes) that drive disease progression [7]. Identification of these distinct molecular mechanisms, primarily categorized into Type 2 (T2)-high and non-T2 inflammation, is essential for the targeted application of biological therapies [8]. Consequently, integrating multi-omic biomarkers with functional data is no longer elective but a fundamental requirement for achieving true precision in respiratory medicine [9].

**Objectives of the Review: Integrating Biological Biomarkers with Functional Parameters.**

**Figure 1:**
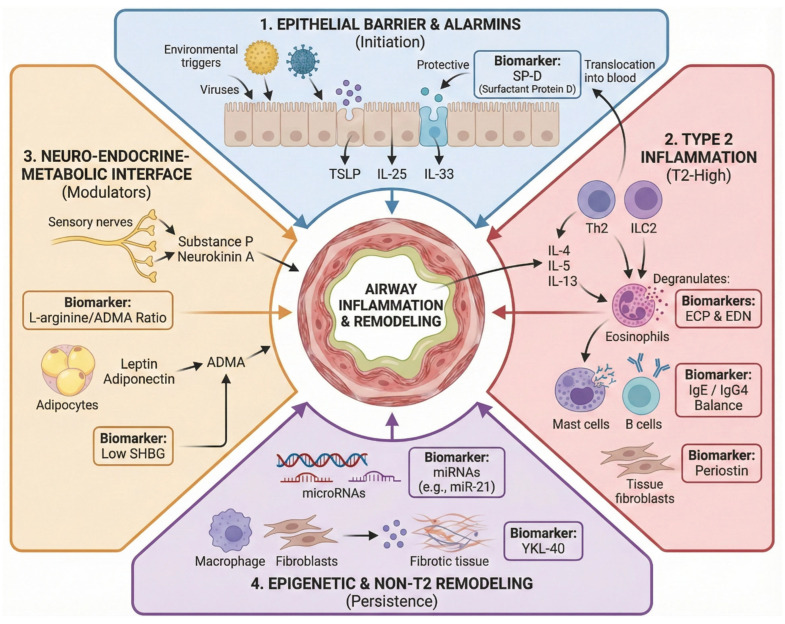
**Multidimensional integration of pathophysiological mechanisms and emerging biomarkers in severe bronchial asthma.** This schematic diagram illustrates the multidimensional pathophysiology of severe bronchial asthma, highlighting the convergence of four distinct biological domains—epithelial initiation, Type 2 immunity, neuro-metabolic modulation, and epigenetic persistence—upon airway inflammation and structural remodeling. The upper and right quadrants depict the classic inflammatory cascade driven by epithelial alarmins and subsequent cellular activation, linking key effector interleukins (IL-4, IL-5, IL-13) alongside specific degranulation and structural biomarkers (ECP, EDN, periostin) to their respective cellular sources. Concurrently, the left and lower sections detail how the neuro-metabolic interface (involving ADMA and neuropeptides) and epigenetic factors (microRNAs) act as systemic modulators to drive disease persistence and alternative remodeling pathways (YKL-40). **Abbreviations:** ADMA (asymmetric dimethylarginine), ECP (eosinophil cationic protein), EDN (eosinophil-derived neurotoxin), IgE (immunoglobulin E), IgG4 (immunoglobulin G4), IL (interleukin (e.g., IL-4, IL-5, IL-13, IL-25, IL-33)), ILC2 (Type 2 innate lymphoid cells), miRNA (microRNA), SHBG (sex hormone-binding globulin), SP-D (surfactant protein D), Th2 (T helper 2 cells), TSLP (thymic stromal lymphopoietin), YKL-40 (chitinase-3-like protein 1).

The primary objective of this review is to provide a comprehensive synthesis of how emerging molecular biomarkers intersect with physiological assessments to refine asthma endotyping, see Figure 1. By evaluating the correlation between Type 2 alarmin cascades, specifically TSLP (thymic stromal lymphopoietin), IL-25, and IL-33, and distal airway resistance measurements, this paper aims to bridge the gap between basic immunology and bedside respiratory mechanics [10,11]. Furthermore, we explore the modulatory role of the neuro-hormonal axis and niche markers like YKL-40 or CHI3L1 (chitinase-3-like protein 1) and SP-D (surfactant protein D), positioning them as essential components of a multidimensional diagnostic framework [12]. Ultimately, this integration seeks to elucidate the pathophysiological drivers of small airway dysfunction, thereby facilitating a more targeted and specific approach to therapeutic intervention in difficult-to-treat asthma [13].

Crucially, this multidimensional diagnostic framework must be viewed through a temporal lens. Asthma pathogenesis is not a static convergence of parallel pathways, but rather a progressive biological continuum. By delineating biomarkers of early, active inflammation (such as ECP and EDN) from those signifying downstream structural consequences and chronic immunometabolic shifts (such as periostin, YKL-40, SP-D, and ADMA), we aim to provide a time-integrated model of disease progression. This distinction is vital for accurately timing therapeutic interventions according to the specific evolutionary phase of the patient’s endotype [11].

Building upon this temporal perspective, our framework extends beyond mere cross-sectional endotyping to encompass longitudinal risk prediction. By highlighting ongoing biological activity within the peripheral airways, we demonstrate how these multi-omic biomarkers can independently predict future exacerbations, accelerated lung function decline, and the progression toward fixed airflow limitation—even when baseline spirometry remains stable [14]. Consequently, this review links endotype-driven stratification not merely to initial biologic therapy selection, but also to proactive risk modification and the long-term management of the disease.

Furthermore, it is important to note that while specific statistical correlations (e.g., values) are cited throughout this narrative review to illustrate biological relationships, these metrics frequently derive from heterogeneous observational studies. Many of these associations remain weak to moderate, and their clinical interpretation must ultimately account for varying sample sizes, study designs, and unadjusted confounders.

## 2. The Immunological Landscape of Asthma: Key Pathogenic Drivers and Molecular Checkpoints

The complex pathophysiology of bronchial asthma is increasingly recognized as a complex interaction between the airway epithelium, innate immune cells, and adaptive Th2-driven responses. Central to this process is the ‘alarmin’ cascade, where epithelial-derived cytokines respond to environmental insults by triggering a downstream inflammatory environment characterized by high-affinity immunoglobulin E (IgE) production and eosinophilic recruitment [15]. Understanding these basic pathways is essential for deciphering the molecular endotypes that dictate clinical severity and responsiveness to biological interventions [16].

### 2.1. Peripheral Eosinophilia and Beyond: ECP and EDN as Precision Markers of Active Degranulation

The persistence of peripheral eosinophilia, despite standard-of-care therapy, is currently recognized as a major predictor for the imminent risk of severe exacerbations, reflecting a biological instability that necessitates rigorous clinical monitoring [17]. But the diagnostic utility of the absolute blood eosinophil count (**BEC**) is increasingly re-evaluated due to its inability to distinguish between non-pathogenic circulating cells and those actively contributing to mucosal injury [18]. From a prognostic and temporal perspective, while the absolute blood eosinophil count remains a validated, static predictor for initial biologic therapy selection, it falls short in mapping the disease over time. BEC merely indicates the systemic potential for Type 2 inflammation; it does not capture the transition to downstream tissue damage. Therefore, relying solely on systemic cell enumeration is insufficient for predicting the imminent risk of remodeling progression or persistent small airway involvement [19]. Although composite indices such as the eosinophil-to-lymphocyte ratio (ELR) in asthma or the neutrophil-to-lymphocyte ratio (**NLR**) in other lung diseases offer valuable prognostic insights regarding systemic inflammation, they do not directly quantify the degranulation status [20,21]. While peripheral eosinophilia serves as a hallmark of Type 2-high asthma, it frequently correlates poorly with the intensity of airway inflammation and the degree of structural remodeling [17,22]. Consequently, the quantification of eosinophil-derived proteins, specifically eosinophil cationic protein (**ECP**) and eosinophil-derived neurotoxin (**EDN**), has emerged as a more accurate reflection of effector cell activation [23]. ECP, a potent ribonuclease, exerts direct cytotoxic effects on the bronchial epithelium, facilitating the breakdown of the mucosal barrier and exacerbating airway hyperresponsiveness [24]. Crucially, this cytotoxic effect is disproportionately amplified within the small airways. While FEV1 may remain stable, ongoing degranulation in the distal lung drives peripheral mucus plugging and subtle luminal narrowing, establishing SAD as a biologically active compartment even in visually ‘controlled’ asthma [23]. Similarly, EDN acts as a robust indicator of the eosinophil’s degranulation status, offering a superior biochemical profile of the inflammatory burden compared to simple cellular enumeration [23,24].

From a temporal perspective, ECP and EDN capture the early, highly active phase of Type 2 biology. Unlike markers of chronic tissue alteration, these proteins reflect the immediate cytokine-driven eosinophilic activation and ongoing mucosal injury. They represent an acute inflammatory activity of the disease, allowing clinicians to detect exacerbation risks before irreversible downstream structural changes occur. Researchers demonstrate a significant inverse correlation between elevated levels of these proteins and FEV1 in asthmatic patients, notably including those with stable BEC (EDN: r = −0.21, *p* < 0.001; ECP: r = −0.48, *p* < 0.001) [25,26]. Furthermore, these biomarkers have demonstrated high sensitivity in predicting impending asthma exacerbations, often preceding the clinical deterioration by several days [27,28]. In the context of precision medicine, these biomarkers provide an objective metric for monitoring the efficacy of biological therapies, such as anti-IL-5 or anti-IL-5R agents. By quantifying the specific release of toxic granules, clinicians can better assess the inflammatory activity of the disease rather than its mere presence [29]. This transition from a quantitative to a qualitative cellular assessment is pivotal for identifying patients who may require intensified corticosteroid therapy or earlier initiation of biologics. Ultimately, integrating ECP and EDN into the diagnostic algorithm bridges the gap between systemic hematology and localized respiratory pathology [30]. Furthermore, the prognostic implications of this localized peripheral airway activity extend far beyond immediate clinical deterioration. Because persistent degranulation in the distal lung operates silently—often independent of baseline FEV1—elevated ECP and EDN serve as crucial predictors of longitudinal risk. They identify a subgroup of patients uniquely predisposed to an accelerated trajectory of lung function decline, allowing clinicians to implement risk modification strategies long before lung function declines on spirometry [31]. This multi-dimensional approach ensures that therapeutic decisions are based on the actual biochemical footprint of the eosinophil within the distal lung compartment [28,29,30].

### 2.2. Humoral Dynamics: IgE as the Primary Biomarker in Allergic Asthma and the IgG4 Isotype Switch

**IgE** remains the main humoral mediator of allergic asthma, orchestrating the immediate hypersensitivity response by cross-linking high-affinity receptors (FcεRI) on mast cells and basophils [32]. However, contemporary research emphasizes that the humoral profile is not defined by IgE alone, but rather by its dynamic interaction with the IgG4 (immunoglobulin G4) isotype. Under the influence of IL-10 (interleukin 10) and chronic allergen exposure, a class-switch recombination toward IgG4 can occur, where these non-inflammatory antibodies act as ‘blocking antibodies’ by competing for allergen binding sites [33]. This isotype switch is clinically significant as it serves as a key biomarker for immune tolerance and the successful modulation of the allergic cascade during allergen-specific immunotherapy. A significant reduction in the IgE/IgG4 ratio is strongly associated with clinical efficacy, evidenced by symptom alleviation and reduced medication use, showing a robust effect size (SMD = 0.81; 95% CI, 0.71–0.91). Conversely, a sustained or elevated IgE/IgG4 ratio correlates with higher combined symptom and medication scores (*r* = 0.23, *p* = 0.04), indicating a lack of protective tolerance and persistent susceptibility to exacerbations. Thus, monitoring the transition from IgE-mediated sensitization to IgG4-related modulation provides a better understanding of the patient’s underlying immune endotype and long-term clinical aspects [34].

## 3. Orchestrating Type 2 Inflammation: The Canonical Roles of Interleukins 4, 5, 13 and Periostin

The pathophysiology of Type 2-high asthma is triggered by a network of key cytokines—**IL-4** (interleukin 4), **IL-5** (interleukin 5), and **IL-13** (interleukin 13)—which serve as the primary drivers of chronic airway inflammation, produced not only from adaptive Th2 lymphocytes but also from type 2 innate lymphoid cells (ILC2s) [15]. IL-4 functions as the critical primary regulator essential for the differentiation of naive CD4^+^ T cells and for driving B cell immunoglobulin class-switch recombination toward IgE, thus initiating the sensitization phase [35]. While IL-4 controls immune programming, IL-5 acts as the final maturation factor and survival signal for eosinophils, preventing their apoptosis and facilitating their recruitment from the bone marrow to the bronchial tissue [36]. Distinctively, IL-13 acts as the dominant effector cytokine governing the physiological phenotype; it induces goblet cell metaplasia, promotes smooth muscle contractility, and notably upregulates inducible nitric oxide synthase (iNOS), a mechanism directly correlating with elevated fractional exhaled nitric oxide (FeNO) levels [37]. Furthermore, the IL-4/IL-13 axis converges on the IL-4 receptor alpha subunit within the Type II receptor complex, activating STAT6 signaling to drive subepithelial fibrosis and airway remodeling [38]. The blockade of these specific pathways by monoclonal antibodies has validated their roles, demonstrating significant reductions in exacerbations and improvements in lung function (FEV 1) in refractory phenotypes [39].

Complementing the previously discussed cytokine profile, particular emphasis must be placed on a biomarker that has garnered significant attention, cited in the ERS/ATS (European Respiratory Society/American Thoracic Society) technical standards as an emerging tool and referenced in the GINA (Global Initiative for Asthma) recommendations [1,40]. **Periostin** is a matricellular protein induced primarily by IL-13 and IL-4 in airway epithelial cells and lung fibroblasts, functioning as a critical mediator of subepithelial fibrosis and extracellular matrix reorganization intrinsic to airway remodeling [41]. Unlike dynamic inflammatory markers, serum periostin serves as a stable surrogate for the localized ‘Type 2-high’ endotype, demonstrating a superior correlation with basement membrane thickening and tissue eosinophilia compared to peripheral blood indices [42]. Temporally, periostin represents a downstream consequence of sustained Type 2 signaling rather than an early inflammatory trigger. While acute cytokines and degranulation products fluctuate dynamically during exacerbations, periostin accumulates as a result of chronic biological progression, serving as a stable long-term marker of prolonged IL-13 exposure and established subepithelial remodeling [41]. Consequently, elevated periostin levels identify a specific patient phenotype characterized by fixed airflow obstruction and steroid resistance, offering significant predictive value for the therapeutic response to biological agents targeting the IL-13/IL-4 axis [43]. From a prognostic standpoint, periostin is not merely a reflection of current remodeling, but a strong predictor of future risk. Sustained elevation of serum periostin strongly predicts a steeper longitudinal decline in lung function and an irreversible progression toward fixed airflow limitation. Incorporating this biomarker into routine assessment thus shifts the therapeutic goal from short-term symptom control to long-term risk modification, aiming to halt structural deterioration in its highly active, even if clinically silent, peripheral stages [41].

## 4. The Epithelial Barrier and Alarmin Cascades: TSLP, IL-25, and IL-33 as Sensors of Mucosal Injury

The airway epithelium is no longer viewed merely as a passive physical barrier, but rather as a metabolically active immune sensor that orchestrates the initiation of the inflammatory response through the release of ‘alarmins’: **TSLP**, **IL-25**, and **IL-33** [44]. Upon exposure to environmental insults—ranging from allergens and pollutants to viral pathogens—epithelial pattern recognition receptors (PRRs) trigger the rapid secretion of these cytokines, which serve as the primary bridge between innate sensing and adaptive immunity [45]. TSLP acts as a broad-spectrum key upstream regulator located upstream of the classic Th2 cascade, conditioning dendritic cells to promote a pro-allergic phenotype even in the absence of specific IgE sensitization [46]. Concurrently, IL-33, constitutively stored within the nucleus of epithelial cells, functions as a classic ‘danger signal’ (DAMP—damage-associated molecular pattern) released instantaneously upon necrotic cell death or mechanical stress to alert the immune system of a barrier breach [47]. These alarmins are the most potent activators of ILC2s, driving the secretion of IL-5 and IL-13 independently of T cell mediation, which explains the persistence of inflammation in non-atopic severe asthma. Furthermore, chronic alarmin signaling perpetuates a cycle of aberrant repair and subepithelial fibrosis, linking epithelial dysfunction directly to the structural remodeling observed in establishing fixed airflow obstruction. Consequently, targeting these upstream mediators—as exemplified by anti-TSLP therapies—offers a mechanism to inhibit the entire inflammatory response at its source, regardless of the downstream eosinophilic or allergic phenotype [48].

## 5. The Neuro-Hormonal Interface: Systemic and Local Modulators of Airway Reactivity

The heterogeneity of bronchial asthma extends beyond classical immune pathways, being significantly modulated by a complex neuro-endocrine interface that dictates both airway tone and structural remodeling [49], see Table 1.

### 5.1. Endocrine Regulation: Sex Steroids and SHBG as Drivers of Immune Polarization (Testosterone vs. Estrogen)

The clear sex differences in adult asthma are largely driven by the opposing effects of sex steroid hormones on Type 2 inflammation. Current research highlights that **testosterone** exerts a protective immunosuppressive role by potently inhibiting ILC2s, thereby reducing the release of IL-5 and IL-13 [50]. In contrast, fluctuating estrogen levels can enhance mast cell releasability, while reduced levels of sex hormone-binding globulin (**SHBG**) are increasingly recognized as a biomarker for the Obese-Asthma phenotype, significantly correlating with lung volumes (e.g., FEV1/FVC ratio (forced expiratory volume in 1 s) and FVC (forced vital capacity): *r* = 0.35, *p* < 0.001) [51].

### 5.2. Neuro-Immune Crosstalk in Asthma: The Pathogenic Imbalance of Bronchoconstrictive and Bronchodilatory Neuropeptides

Dysregulation of airway neuronal control constitutes a central pathogenic factor in bronchial asthma, where the peripheral nervous system actively modulates both innate and adaptive immune responses through the local release of neurotransmitters that sustain airway hyperresponsiveness [52]. This complex neuro-immune interface transforms allergen exposure into a continuous inflammatory cascade, wherein airway nociceptors function as critical regulators of type 2 immunity and clinical symptomatology, including cough and mucous hypersecretion [53]. Consequently, targeting these neural pathways has emerged as a promising therapeutic strategy, shifting the paradigm from exclusively anti-inflammatory treatments to comprehensive neuromodulatory interventions designed to interrupt the amplification of the neuro-allergic loop [54].

**Figure 2:**
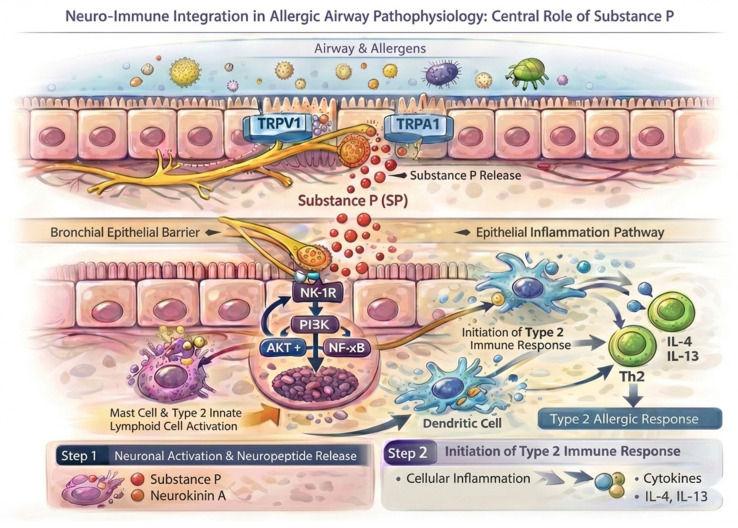
**The neuro-immune interface: mechanisms of tachykinin-mediated Type 2 airway inflammation.** This schematic illustrates the neuro-immune crosstalk within the asthmatic airway, demonstrating how environmental triggers activate sensory nerve endings via TRPV1 and TRPA1 channels to release pro-inflammatory neuropeptides, predominantly substance P. Once released, substance P binds to neurokinin-1 receptors (NK-1R) on effector cells such as mast cells and innate lymphoid cells, initiating intracellular signaling cascades (PI3K/AKT/NF-κB) that drive cellular activation. Ultimately, this neurogenic mechanism amplifies the classical allergic pathway by promoting dendritic cell engagement and stimulating Th2 lymphocytes to secrete key Type 2 cytokines (IL-4, IL-13), thereby sustaining chronic airway inflammation. **Abbreviations: TRPV1** (Transient Receptor Potential Vanilloid 1), **TRPA1** (Transient Receptor Potential Ankyrin), SP (substance P), **NK-1R** (neurokinin 1 receptor), **PI3K** (phosphoinositide 3-kinase), **AKT+** (protein kinase B / Akt), **Th2** (T helper 2 cell), **IL-4** (interleukin-4), **IL-13** (interleukin-13).

At the molecular level, this mechanism is governed by the activation of transient receptor potential channels (particularly TRPV1 and TRPA1) on sensory nerve endings, which triggers the antidromic release of pro-inflammatory tachykinins, predominantly **substance P** and **neurokinin A** [55,56,57], see Figure 2. These neuropeptides exert direct effects upon NK1 receptors (NK-1R) located on mast cells and type 2 innate lymphoid cells (ILC2s), stimulating degranulation and cytokine production—a phenomenon significantly exacerbated by a concomitant deficit of **vasoactive intestinal peptide (VIP)**, the primary bronchodilator of the non-adrenergic non-cholinergic (NANC) system [58]. Thus, an altered ratio favoring substance P-mediated constriction over VIP-mediated relaxation acts as the primary driver for persistent neurogenic inflammation and airway remodeling, underscoring the potential of these neuropeptides as specific prognostic biomarkers.

### 5.3. The Metabolic Nexus: Adipokines, ADMA, and the Mechanism of Nitric Oxide Uncoupling in Obese Asthma

The intersection of metabolic dysregulation and airway inflammation defines a distinct “obese-asthma” endotype, driven by pathobiological mechanisms that transcend classical Type 2 immunity [59]. Central to this phenotype is the systemic imbalance of adipokines (**leptin** and **adiponectin**), characterized by hyperleptinemia and hypoadiponectinemia, which has been shown to correlate strongly with asthma severity independent of BMI (body mass index)(*r* = 0.45, *p* < 0.001) [60]. This pro-inflammatory metabolic environment upregulates protein arginine methyltransferases, leading to the significant accumulation of asymmetric dimethylarginine (**ADMA**) in both serum and lung tissue, acting as a potent endogenous inhibitor of nitric oxide synthase (NOS) [61]. Crucially, in the presence of elevated ADMA, airway NOS becomes “uncoupled,” shifting its enzymatic activity from producing bronchodilatory nitric oxide (NO) to generating cytotoxic superoxide anions and peroxynitrite [62]. This unchecked oxidative stress and localized endothelial dysfunction severely impact the dynamic immunometabolic interface of the small airways. The resulting peripheral resistance and distal air trapping highlight how metabolic dysregulation specifically fuels SAD, perpetuating a silent clinical decline that macroscopic functional tests fail to detect. This oxidative shift explains the clinical paradox where obese asthmatics frequently exhibit low fractional exhaled NO (**FeNO**) levels despite active inflammation, rendering traditional biomarkers falsely negative in this population [59]. Within the time-integrated progression of asthma, this ADMA-related NOS uncoupling should be conceptualized as a later immunometabolic shift rather than a primary inflammatory trigger. It reflects a chronic evolutionary phase where prolonged systemic metabolic dysregulation—driven by adipose tissue—permanently alters the local airway microenvironment, shifting the pathobiology from an early reactive state to a persistent, structurally consolidated, and steroid-resistant phenotype [63]. Cohort analyses have demonstrated that a reduced L-arginine/ADMA ratio serves as a superior predictor of lower FEV1 and poorer asthma control compared to serum IgE or eosinophils in late-onset metabolic phenotypes (*p* < 0.01) [64]. Furthermore, the oxidative stress induced by NOS uncoupling contributes directly to corticosteroid resistance, a hallmark challenge in treating obese asthmatics [65]. ADMA acts as a central metabolic checkpoint, effectively bridging systemic adiposity with local airway endothelial dysfunction and structural remodeling. Recognizing this “Metabolic Nexus” shifts the therapeutic perspective from broad immunosuppression to the targeted restoration of NO bioavailability. Thus, the L-arginine/ADMA ratio emerges not merely as a biomarker of endothelial dysfunction, but as a critical stratifying tool for identifying patients who may benefit from metabolic interventions rather than dose-escalated steroids [66]. Ultimately, dissecting this pathway validates the necessity of integrating metabolic profiling into the precision management of severe, non-atopic asthma.

## 6. Emerging Biomarkers: Transcending the Classical Paradigm in Asthma Pathogenesis

### 6.1. The Epigenetic Landscape: MicroRNAs as Epigenetic Regulators of Therapeutic Heterogeneity

The clinical trajectory of severe asthma is frequently affected by epigenetic modifications that orchestrate therapeutic heterogeneity, with microRNAs (miRNAs) serving as the key modulators of post-transcriptional gene regulation [67]. Unlike static genomic polymorphisms, the miRNA landscape is dynamic and responsive to environmental stimuli; specifically, the dysregulation of the **miR-21** axis has emerged as a central driver of corticosteroid insensitivity in severe phenotypes [68]. Mechanistically, miR-21 targets phosphatase and tensin homolog (PTEN) and histone deacetylase 2 (HDAC2), thereby blocking the anti-inflammatory effects of glucocorticoids even at high dosages [68]. In comparative cohort studies involving severe asthmatics, serum miR-21 levels exhibited a significant inverse correlation with FEV1 (*p* < 0.001) and were markedly elevated in steroid-resistant patients compared to sensitive controls [69,70]. When contextualized within our time-integrated prognostic framework, the insufficiency of peripheral eosinophil counts becomes even more apparent in these complex phenotypes. While blood eosinophils may guide early biologic intervention, epigenetic regulators like miR-21 are critical for proactive risk stratification. They identify patients on a dangerous longitudinal trajectory—those at high risk for entrenched corticosteroid resistance, aggressive remodeling progression, and persistent small airway disease—even when conventional systemic inflammatory markers appear deceptively stable [71]. Beyond steroid resistance, **miR-146a** and **miR-155** function as central molecular regulators of Type 2 inflammation [72,73]. Reduced expression of miR-146a, a potent negative regulator of the NF-κB pathway, is consistently observed in the airway smooth muscle of severe patients, statistically correlating with increased exacerbation frequency and hospitalization rates (*p* < 0.05). Furthermore, variations in the let-7 miRNA family modulate the IL-13 downstream signaling pathway, offering a plausible molecular explanation for why patients with identical blood eosinophil counts respond disparately to anti-IL-5 or anti-IL-4 biologics. In the quest for diagnostic precision, seminal transcriptomic profiling has identified robust circulatory signatures capable of differentiating asthma phenotypes. Recent advances highlight the specific potential of circulating microRNAs not only as diagnostic tools but also as critical indicators of severe asthma risk [69]. Through sophisticated gene network analyses, researchers are increasingly able to map complex miRNA interactions, identifying novel biomarker candidates that reflect intrinsic airway pathology [74]. For instance, specific panels comprising markers like miR-125b, miR-16, miR-299-5p, miR-155, and miR-570 possess the unique capacity to differentiate asthmatic patients from those with allergic rhinitis—a distinction often obscured by overlapping atopic features [75]. Furthermore, comprehensive profiling studies underscore the broader therapeutic and diagnostic implications of the miRNA landscape in respiratory diseases, validating their utility as dynamic indicators of physiological impairment, disease severity, and lung function decline [76].

These small non-coding RNAs do not merely reflect disease activity but actively perpetuate airway remodeling through the regulation of epithelial–mesenchymal transition [77]. Moreover, the remarkable stability of miRNAs in peripheral blood facilitates “liquid biopsy” approaches, allowing for non-invasive monitoring of inflammation dynamics [78]. Consequently, profiling these epigenetic markers provides a higher-resolution stratification tool than classical cytokines, shifting the paradigm from reactive symptom management to predictive precision medicine.

### 6.2. YKL-40: A Molecular Bridge Between Neutrophilic Inflammation and Structural Remodeling

**YKL-40**, biologically classified as a chitinase-like protein (CLP) that retains chitin-binding capability but lacks enzymatic hydrolytic activity, functions as a critical tissue growth factor and inflammatory mediator within the lung microenvironment [79]. In the context of bronchial asthma, its upregulation is intrinsically linked to structural remodeling and subepithelial fibrosis, distinguishing it from classical cytokines by its specific release from macrophages and epithelial cells rather than lymphocytes. Clinical studies conducted over the last decade have consistently demonstrated a statistically significant negative correlation between serum YKL-40 levels and forced expiratory volume in 1 s (FEV1), establishing it as a robust marker of fixed airflow obstruction and disease severity [80]. Furthermore, elevated circulating concentrations have been positively associated with total IgE levels (*r* = 0.528, *p* < 0.001) and exacerbation severity, while demonstrating a significant negative correlation with lung function parameters (*r* = −0.529, *p* < 0.001), thus serving as a robust marker for accelerated functional decline [81]. Fundamentally, contemporary research is increasingly positioning YKL-40 as a defining biomarker for the ‘non-eosinophilic’ or neutrophilic asthma phenotypes, a subgroup often characterized by corticosteroid resistance and normal type 2 inflammatory markers [82]. This distinct profile suggests that YKL-40 drives an alternative pathway of inflammation and fibrosis, making it an invaluable candidate for stratifying patients who fall outside the therapeutic scope of traditional biologics.

### 6.3. Beyond Surface Tension: Surfactant Protein D as the Regulator of the Muco–Microbial Interface

Surfactant protein D (**SP-D**) has expanded beyond its classical role in surface tension regulation to emerge as a important soluble pattern recognition receptor (PRR) essential for modulating pulmonary immune homeostasis [83]. Functioning as a “molecular mediator” between innate immunity and the commensal microbiota, SP-D binds to microbial carbohydrate ligands via its carbohydrate recognition domain (CRD), thereby facilitating pathogen clearance while simultaneously suppressing T2-driven allergic inflammation [84]. However, in asthmatic patients, this protective shield is frequently compromised; studies indicate that specific single nucleotide polymorphisms (SNPs) in the *SFTPD* gene, such as the Met11Thr variant, lead to the assembly of structurally defective multimers with reduced ligand-binding affinity [85]. This structural impairment disrupts the lung microbiome equilibrium, permitting the expansion of pathogens like *Haemophilus influenzae*, which further drives neutrophilic inflammation and steroid resistance. Clinically, serum SP-D serves as a robust biomarker of epithelial integrity loss; unlike healthy states where SP-D is confined to the alveoli, asthmatic inflammation induces a significant translocation of SP-D into the systemic circulation. Quantitative analyses reveal that serum SP-D levels are significantly elevated in acute asthma exacerbations compared to stable controls (*p* < 0.001), exhibiting a strong inverse correlation with FEV 1 (*r* = −0.42, *p* < 0.01) [86,87]. Conversely, reduced levels of functional SP-D within the bronchoalveolar lavage (BAL) are associated with a heightened susceptibility to viral-induced exacerbations, particularly rhinovirus infections, highlighting a failure in the innate antiviral defense [88]. Reflecting the cumulative burden of mucosal leakage and inflammation, serum SP-D levels serve as an independent predictor of exacerbations [89]. Furthermore, the interaction between SP-D and alveolar macrophages is crucial; defective signaling shifts macrophage polarization towards a pro-inflammatory M1 phenotype, perpetuating the cycle of tissue damage. Thus, quantifying SP-D offers dual utility: it assesses the functional competence of the lung’s “innate shield” against dysbiosis and serves as a circulating metric of epithelial barrier permeability. Integrating SP-D profiling into the biomarker panel provides a unique window into the infectious and structural components of asthma pathogenesis that eosinophils alone cannot reveal [83].

## 7. Clinical Readiness and the Translational Gap

While multi-omic profiling offers profound insights into asthma pathophysiology, the translation of these biomarkers into routine clinical practice varies significantly. In daily medical care, only a select few biomarkers possess the regulatory approval and robust validation required for broad adoption. Clinically established markers, specifically the absolute BEC, FeNO, and IgE, constitute the current standard of care. These markers benefit from highly standardized and cost-effective assays and are supported by extensive data from large randomized controlled trials. Consequently, they serve as the primary biomarkers for selecting approved biologic therapies [1]. Nevertheless, they present well-documented limitations; for instance, BEC merely reflects systemic inflammation and lacks tissue-level specificity, while FeNO is frequently confounded by obesity or smoking [90,91,92], see Table 2.

To address these diagnostic gaps, a second tier of emerging biomarkers is being actively evaluated. Molecules such as eosinophil cationic protein (ECP) and eosinophil-derived neurotoxin (EDN) provide a more accurate biochemical measure of local eosinophil activation compared to simple systemic cell counts [26,29]. Similarly, serum periostin was initially proposed as a highly stable surrogate for airway remodeling. Despite their strong physiological rationale, the large-scale clinical adoption of these emerging markers remains limited [23]. For example, periostin’s utility for clinical stratification has declined recently following inconsistent validation studies and interference from confounding factors like bone metabolism [93]. Currently, ECP and EDN testing requires specific sample handling and lacks universally accepted clinical cut-offs [23,93]. Finally, the most complex indicators discussed in this review operate almost exclusively within the experimental or research-only domain. Biomarkers such as microRNAs, surfactant protein D (SP-D), YKL-40, and asymmetric dimethylarginine (ADMA) provide exceptional value in understanding steroid resistance, small airway disease, and metabolic phenotypes. However, current evidence for these molecules relies predominantly on small observational cohorts, transcriptomic profiling, or animal models. Their analytical methods are highly specialized, lack global standardization, and remain too expensive for everyday healthcare [61,78,82,89]. The concept of miRNA “liquid biopsies,” while theoretically transformative, remains strictly investigational and far from immediate clinical application [75]. Transitioning these experimental markers into practical diagnostic tools requires large-scale prospective validation, rigorous assay standardization, and clear cost-effectiveness analyses.

## 8. Conclusions and Future Perspectives

The comprehensive analysis of current literature illustrates that severe asthma is not a singular disease entity but a multifactorial pathophysiological process driven by intricate, overlapping endotypes. The complexity of the evidence reviewed herein reveals that classic Type 2 inflammation frequently intersects with metabolic dysregulation, neurogenic imbalances, and epigenetic modifications, creating a highly complex and individualized biological profile for each patient. In real-world clinical scenarios, these phenotypes exist as a continuum where distinct molecular pathways converge to drive disease persistence. Crucially, this multifaceted profile evolves dynamically over time. The biological progression of asthma transitions from early, cytokine-driven cellular activation (reflected by ECP and EDN) to downstream structural and barrier-related consequences (indicated by periostin, YKL-40, and SP-D), ultimately culminating in late-stage immunometabolic shifts such as ADMA-induced NOS uncoupling. Acknowledging this time-integrated progression is paramount; treating a late-phase metabolic consequence with therapies exclusively targeted at early inflammatory triggers inevitably leads to the ‘functional-symptomatic dissociation’ frequently encountered in severe cohorts.

From a diagnostic standpoint, reliance on a single indicator has proven insufficient. While biomarkers like ECP and EDN provide a detailed assessment of active eosinophilic degranulation, serum periostin offers a stable surrogate for subepithelial fibrosis. Simultaneously, the physiological picture is complicated by metabolic and structural markers, such as ADMA and YKL-40, which elucidate pathways of oxidative stress and non-eosinophilic remodeling invisible to standard inflammatory panels. Moreover, the loss of epithelial integrity, signaled by SP-D and alarmins like TSLP, highlights the failure of the mucosal barrier as an initiating event. Underlying these mechanisms, the epigenetic landscape, governed by specific miRNAs, acts as an epigenetic regulator of steroid resistance and therapeutic heterogeneity, see Table 1.

Fundamentally, the successful transition to precision medicine hinges on explicitly including small airway disease (SAD) within this endotype-driven framework. The biomarkers discussed—from targeted alarmins to metabolic uncouplers—find their primary pathophysiological site within the distal lung. Recognizing SAD as a critical, biologically active disease compartment ensures that therapeutic strategies are not misguided by the apparent stability of preserved FEV1 metrics, but are instead based on true molecular and distal functional realities. Ultimately, the translational relevance of deep phenotyping lies in its prognostic power. Precision medicine must evolve from providing a cross-sectional profile of the patient’s endotype to predicting their disease progression over time. Recognizing that subclinical biological activity within the small airways dictates future risk—often entirely independent of baseline spirometry—empowers clinicians to transition from a reactive model to proactive risk modification, fundamentally altering the progression toward fixed airflow limitation.

However, a significant translational gap remains. While markers such as absolute eosinophil counts and IgE possess established clinical utility for biologic selection, the majority of the advanced molecular indicators discussed herein (e.g., miRNAs, SP-D, ADMA) are still in experimental or early validation stages. Consequently, they are currently unavailable in routine clinical practice.

## Figures and Tables

**Table 1:** **Integrated biomarker profile: from molecular mechanisms to clinical utility.** This table provides a comprehensive classification of asthma biomarkers across diverse pathophysiological domains, including Type 2 inflammation, neuro-endocrine interfaces, and epigenetic regulation. By explicitly linking each biomarker’s underlying molecular mechanism to its clinical relevance, it establishes an integrated framework for deep phenotyping and precision medicine in asthma.

Category/Axis	Biomarker	Pathophysiological Mechanism	Function and Clinical Relevance
**Eosinophilic** **Degranulation**	**ECP** (Eosinophil Cationic Protein)	Direct cytotoxicity on bronchial epithelium; effector phase marker	Indicator of “inflammatory activity”
	**EDN** (Eosinophil-derived Neurotoxin)	Eosinophil activation; reflects actual inflammatory burden better than cell count	Therapeutic monitoring tool
**Humoral Dynamics**	**IgE**	Immediate hypersensitivity; initiates the inflammatory cascade in atopic/extrinsic asthma	Primary allergy mediator
	**IgG4**	Immune tolerance; acts as a “blocking antibody” (competing with IgE)	Tolerance marker (immunotherapy)
	**IgE/IgG4 Ratio**	Balance between sensitization and tolerance	Predictor of immunotherapy efficacy
**Type 2 Inflammation**	**IL-4**	Th2 differentiation and class-switching to IgE	Major target for biological therapies aimed at reducing exacerbations
	**IL-5**	Eosinophil maturation, recruitment, and survival	Major target for biological therapies
	**IL-13**	Goblet cell metaplasia, hyperresponsiveness, iNOS induction	Phenotypic effector
	**Periostin**	Subepithelial fibrosis and remodeling (induced by IL-13/IL-4)	Stable remodeling marker
**Epithelial Barrier (Alarmins)**	**TSLP, IL-25, IL-33**	Innate response to mucosal injury and ILC2 activation	Mucosal injury sensors Major targets for biological therapies (e.g., TSLP)
**Neuro-endocrine and Metabolic Interfaces**	**Testosterone**	Inhibits ILC2 cells, reducing IL-5/IL-13 release	Protective factor
	**SHBG** (Sex Hormone-Binding Globulin)	Regulates bioavailability of free sex steroids.	Marker for Obese-Asthma phenotype
	**Adipokines** (Leptin/Adiponectin)	Pro-inflammatory leptin overwhelms anti-inflammatory adiponectin regulation.	Systemic inflammation induced by adipose tissue
	**ADMA** (Asymmetric dimethylarginine)	Competitive inhibition of NOS; uncoupling of NO production	Cause of the low FeNO paradox
	**Substance P/Neurokinin A**	Neurogenic inflammation; constriction mediated by NK-1 receptors.	Hyperresponsiveness promoters
	**VIP** (Vasoactive Intestinal Peptide)	NANC system (non-adrenergic non-cholinergic)	Natural bronchodilator
**The Epigenetic Landscape (microRNAs)**	**miR-21**	Inhibition of PTEN and HDAC2 (histone deacetylase)	Driver of steroid resistance
	**miR-146a**	Negative regulation of the NF-κB pathway	Inflammatory rheostat
	Circulatory Signature (e.g., **miR-125b**, **miR-299-5p**)	Transcriptomic profiling	Differentiates asthma from allergic rhinitis (liquid biopsy)
**Remodeling & Innate Immunity**	**YKL-40** (Chitinase-like protein)	Tissue proliferation and fibrosis; neutrophilic inflammation	Marker of neutrophilic asthma
	**SP-D** (Surfactant Protein D)	Immune homeostasis and antimicrobial barrier	Marker of epithelial integrity

**Abbreviations: ADMA**, asymmetric dimethylarginine; **ECP**, eosinophil cationic protein; **EDN**, eosinophil-derived neurotoxin; **FcεRI**, high-affinity immunoglobulin E receptor; **FeNO**, fractional exhaled nitric oxide; **FEV1**, forced expiratory volume in 1 s; **FVC**, forced vital capacity; **HDAC2**, histone deacetylase 2; **Ig**, immunoglobulin; **IL**, interleukin; **ILC2**, group 2 innate lymphoid cells; **iNOS**, inducible nitric oxide synthase; **NANC**, non-adrenergic non-cholinergic; **NF-κB**, nuclear factor kappa-light-chain-enhancer of activated B cells; **NO**, nitric oxide; **NOS**, nitric oxide synthase; **PTEN**, phosphatase and tensin homolog; **SHBG**, sex hormone-binding globulin; **SMD**, standardized mean difference; **SP-D**, surfactant protein D; **Th2**, T helper 2 cells; **TSLP**, thymic stromal lymphopoietin; **VIP**, vasoactive intestinal peptide; **YKL-40**, chitinase-3-like protein 1.

**Table 2:** **Critical stratification of asthma biomarkers based on clinical readiness, level of evidence, and accessibility.** This table summarizes the critical stratification of asthma biomarkers into clinically established, emerging, and experimental categories, evaluating them based on their current clinical utility, level of evidence, and assay accessibility.

Biomarker Category	Specific Biomarkers	Current Clinical Status	Level of Evidence and Validation	Assay Standardization and Accessibility
Clinically Established	IgE (Total and Specific); FeNO; Blood Eosinophil Count.	Standard of Care	High	High
Emerging	ECP; EDN; Periostin.	Promising Use	Moderate-to-High	Moderate
Experimental	ADMA; SHBG; SP-D; YKL-40; miRNAs.	Research-only	Low-to-Moderate	Low

**Abbreviations: ADMA:** asymmetric dimethylarginine, **ECP:** eosinophil cationic protein, **EDN:** eosinophil-derived neurotoxin, **FeNO:** fractional exhaled nitric oxide, **IgE:** immunoglobulin E, **miRNAs:** microRNAs, **SHBG:** sex hormone-binding globulin, **SP-D:** surfactant protein D, **YKL-40:** chitinase-3-like protein 1.

## Data Availability

No new data were created or analyzed in this study. Data sharing is not applicable to this article.

## Abbreviations

ADMA	Asymmetric Dimethylarginine
ATS	American Thoracic Society
BAL	Bronchoalveolar Lavage
BEC	Blood Eosinophil Count
BMI	Body Mass Index
CD4+	Cluster of Differentiation 4 (T helper cells)
CI	Confidence Interval
CLP	Chitinase-like Protein
CRD	Carbohydrate Recognition Domain
DAMP	Damage-Associated Molecular Pattern
ECP	Eosinophil Cationic Protein
EDN	Eosinophil-Derived Neurotoxin
ELR:	Eosinophil-to-lymphocyte ratio
ERS	European Respiratory Society
FcεRI	High-affinity IgE receptor
FeNO	Fractional Exhaled Nitric Oxide
FEV1	Forced Expiratory Volume in 1 s
FVC	Forced Vital Capacity
GINA	Global Initiative for Asthma
HDAC2	Histone Deacetylase 2
IgE	Immunoglobulin E
IgG4	Immunoglobulin G4
IL	Interleukin (e.g., IL-4, IL-5, IL-13, IL-25, IL-33)
IL-5R	Interleukin-5 Receptor
ILC2s	Innate Lymphoid Cells type 2
iNOS	Inducible Nitric Oxide Synthase
IOS	Impulse Oscillometry
miRNAs	MicroRNAs
NANC	Non-Adrenergic Non-Cholinergic
NF-B	Nuclear Factor kappa B (standard: NF-κB)
NK-1R	Neurokinin-1 Receptor
NLR	Neutrophil-to-lymphocyte ratio
NO	Nitric Oxide
NOS	Nitric Oxide Synthase
OR	Odds Ratio
PRRs	Pattern Recognition Receptors
PTEN	Phosphatase and Tensin Homolog
SFTPD	Surfactant Protein D Gene
SHBG	Sex Hormone-Binding Globulin
SMD	Standardized Mean Difference
SNPs	Single Nucleotide Polymorphisms
SP-D	Surfactant Protein D
STAT6	Signal Transducer and Activator of Transcription 6
T2	Type 2 (inflammation)
Th2	T helper 2
TRPA1	Transient Receptor Potential Ankyrin 1
TRPV1	Transient Receptor Potential Vanilloid 1
TSLP	Thymic Stromal Lymphopoietin
VIP	Vasoactive Intestinal Peptide
YKL-40	Chitinase-3-like protein 1 (Human Cartilage Glycoprotein-39)
